# Supplementary material for: Are carbon emissions trading and green financial instruments synergistic? -Comprehensive quantitative research based on content analysis
Source: PLoS One. 2024 Mar 7;19(3):e0298601. doi: 10.1371/journal.pone.0298601 (PMC10919601; doi:10.1371/journal.pone.0298601)
Supplement: S1 Data — (ZIP) [file pone.0298601.s001.zip › Data/地级市Z_F工作报告.zip!/╔╜╢1⁄2/┴┘╥╩╩╨/└┤╫╘╣1⁄2╓┌║┼í╛┬φ┐╦╩2╛▌═°í┐_2018.docx]

各位代表：

现在，我代表市人民政府向大会报告工作，请予审议，并请市政协委员和列席会议的同志提出意见。

一、2017年工作回顾

刚刚过去的2017年，是很不平凡的一年。面对错综复杂的国内外形势和我国经济发展进入新常态等一系列深刻变化，全市上下在市委的坚强领导下，坚持以习近平新时代中国特色社会主义思想为指导，认真贯彻落实中央和省委、省政府决策部署，按照“走在前列”的目标定位，聚焦聚力抓转型，攻坚克难促规范，统筹推进改革发展稳定各项工作，较好地完成了市十九届人大一次会议确定的各项任务，“大美新”临沂建设取得新的成就。

(一)产业转型升级有了新进展。围绕建设现代产业新城，加快存量变革、增量崛起，实体经济发展提质增效。预计全市生产总值达到4500亿元，增长8%左右；实现一般公共预算收入285.3亿元，同口径增长3.9%，剔除减税降费、清理一次性欠税等因素，实际增长10%。工业加快向“中高端”迈进。综合运用“三个工具”淘汰落后产能，累计清理取缔“散乱污”企业8460家，淘汰钢铁产能120万吨、化工产能220万吨、非煤矿山产能900万吨，改造提升建陶生产线1.2亿平方米，解决了一大批污染重、有安全生产隐患的老大难问题。坚持“一业一策”，制定和落实木业、食品、医药、建材等传统产业转型升级方案，实施过亿元技改项目650个，全市规模以上工业总产值达到1.1万亿元、增长9.9%，万元GDP能耗下降9%左右。坚持引进孵化转化“一齐抓”，强化创新驱动，培育新兴产业。新认定国家级高新技术企业67家，高新技术产业产值占比达到29.5%。新建省级以上创新平台47家，新增产学研合作企业615家。经开区被评为2017年中国产业园区创新力百强，高新区电子元器件及其功能材料产业集群被列入国家试点，河东区获批国家知识产权强县工程示范区，罗欣药业和史丹利获得国家技术发明二等奖，鲁南制药荣获省长质量奖，中印软件园入选国家国际科技合作基地，天元集团成为全国首家“双特五甲”地方企业。侯卫东官场笔记持续深化企业改革，国有和规模以上集体、民营企业规范化改制大部分完成。服务业加快向“新业态”拓展。临沂商城国际化迈出坚实步伐，市场交易额达到4551亿元、增长20%。全市进出口额、物流总额、电子商务交易额分别达到680亿元、2.8万亿元和2930亿元，分别增长17%、7%和35%。通关一体化监管模式落地见效，临沂航空口岸获批对中外籍货运飞机开放，“市场采购”贸易出口60亿元。国际博览中心建成启用，传化、林安等一批商贸物流项目落户国际商贸城。老商贸城市场改造完成116万平方米，瓜达尔等12处海外临沂商城建设进展顺利，京东、亚马逊等一批国际电商服务商登陆临沂，沂采通、沂商通等电商平台上线运行，国家级网络市场监管与服务示范区创建工作在全省率先启动，蒙阴、平邑、沂南入选国家电子商务进农村综合示范县。综合保税区窗口作用有效发挥，进出口总额突破10亿美元。全域旅游扎实推进，全年共接待游客6800万人次，旅游消费总额达到710亿元，分别增长10%和12%。会展经济蓬勃发展，共举办商博会、全国秋季五金商品交易会等各类展会170场次。农业加快向“新六产”发展。提升优质农产品基地106万亩，创建省级以上农产品质量安全县6个、全国平安渔业示范县1个，莒南袁隆平超级稻攻关项目创全省最高单产记录。新认证“三品一标”产品58个，蒙阴蜜桃等15个品牌入围2017中国果品区域公用品牌价值榜，以兰陵蔬菜产业博览会为平台的“产自临沂”农产品品牌越来越响。实施“新六产”示范项目6个，省级以上农业产业化重点龙头企业达到68家，沂南朱家林田园综合体被确定为全省唯一的国家级试点。狠抓“三引一促”，引建项目1231个，到位市外资金1460亿元，引进“千人计划”专家、“泰山学者”等高层次人才238名，带资金、带技术、带项目的创新创业人才3382名，华为科技、华大基因、新松机器人等一批战略项目落户，富士电梯、祎禾科技等一批重大项目投产。成功举办第四届资本交易大会，签约金融招商项目36个、合同金额1238亿元。新引进金融服务机构8家，全市金融机构本外币存贷款余额分别达到5878亿元和4485亿元。

(二)城乡统筹发展形成新局面。围绕建设鲁南苏北区域性中心城市，大力推进中心城区、县城、小城镇和农村新型社区“四级联动”，户籍和常住人口城镇化率分别达到47.6%和57.6%，中心城区建成区人口和面积分别发展到220万人、231平方公里，实现农业转移人口市民化35万人。中心城区建设“提质”。新一轮城市空间发展战略研究全面启动，高铁片区、轨道交通等17个专项规划编制完成，《临沂市城乡规划条例》正式实施。年初确定的288个中心城区建设项目，已完成投资119亿元。北京东路改扩建工程通车，北疏港公路、岚济路大修及临沂机场改扩建等工程竣工。22段“黑臭水体”整治基本完成，沂河河湾和袁家口子水源工程建成蓄水，新建海绵城市28平方公里，集中供热、管道燃气、公共供水普及率分别达到71%、93%和73%。城市管理规范化、精细化、信息化水平进一步提高。县城建设“提效”。新改造棚户区3万户、整治老旧小区251万平方米。兰陵、沂水被确定为省中等城市试点，郯城创建成为国家园林县城，兰陵、费县成为国家卫生县城。特色镇建设“提速”。引导和支持新市镇、重点镇产城融合、镇园合一，因地制宜发展乡村旅游、民俗文化等优势产业，培育产业强镇、经济重镇。蒙阴岱崮、费县许家崖入选全国特色小镇，平邑仲村、沂水马站、郯城新村等被评为省级特色小镇和宜居小镇。

美丽乡村建设“提标”。持续改善农村人居环境，新建、改建农村公路1289公里，建设农村无害化厕所40万户，升级村庄电网754个，实施农村危房改造8300余户，解决了25.8万农村人口的饮水安全问题。新创建省级美丽乡村示范村45个，新增省级传统村落11个，全国“四好农村路”养护现场会、全省美丽乡村建设现场会在我市召开，“好山好水好风情、美丽乡村看沂蒙”城市名片更加亮丽。

(三)生态文明建设取得新成效。围绕建设全国生态文明先行示范区，坚持综合整治和专项治理并重、常态治理和集中整治结合、辖区治污和区域共治联动，着力提升生态环境质量。以中央环保督察为契机，突出抓好工业污染治理、扬尘管控、散煤整治和秸秆禁烧及综合利用等工作，持续铁腕治气，空气质量综合指数同比改善6%以上。在全省率先建立四级河长体系，强化控源截污、垃圾清理、清淤疏浚、生态修复等综合治水措施，13个国省控考核断面水质全部达标，省级以上水利风景区达到30家，沂河入选全省唯一国家级“最美家乡河”，我市被评为全国首批水生态文明城市。全面加强土壤污染防治，危废产生和经营单位规范化管理随机抽查合格率分别达到96%和98%。扎实开展矿山地质修复，完成18个矿山地质环境治理与保护项目，国家级绿色矿山和地质公园达到7家。实施“绿满沂蒙”行动，新增造林面积20万亩、绿地面积753万平方米，森林覆盖率、城市绿化覆盖率分别达到36.2%和41.6%。

(四)人民群众生活得到新改善。围绕增进民生福祉，集中

力量实施十大领域百项惠民工程，全市财政用于民生支出486亿元、占一般公共预算支出的82%，城镇和农村居民人均可支配收入分别达到33266元和12613元，分别增长7.8%和8.3%。大力推进精准脱贫，14.2万贫困人口实现脱贫。扩大就业创业，新增城镇就业12.6万人，转移农村劳动力就业12万人。加强社会保障，企业退休人员基本养老金连续13年提高，退役士兵和权益保障政策全面落实，城乡低保和农村特困人员合规费用医疗救助比例均超过70%，1万余名困难职工和5.6万余名残疾人得到帮扶救助，惠民殡葬改革走在全国前列。均衡发展各类教育，化解“大班额”、全面改薄等工作稳步推进，新建改扩建学校、幼儿园573所，智慧教育云平台惠及学校960所、受益师生80余万人。合理配置医疗资源，二级以上医院医联体建设实现全覆盖，基层医疗卫生机构标准化建设达标率超过90%，49家公立医院规定药品全部零差价销售。积极发展文体事业，新建社区综合文化服务中心3332个、文化小广场5260个，新推出舞台剧《巍巍大青山》等一批文艺精品。持续开展安全生产“大快严”行动，安全生产事故起数、死亡人数同比分别下降44%和29%。不断完善食品药品监管体系，食安山东品牌达到704个。全面加强平安临沂建设，推行基层基础网格实体化、管理员专职化，深入开展排查安全隐患防范四类风险专项行动，圆满完成党的十九大安保维稳任务，全国“雪亮工程”建设推进会在临沂召开，我市被评为全国社会治安综合治理优秀市，市公安局被表彰为全国社会治安综合治理先进集体。

(五)政府自身建设出现新气象。围绕建设有为、高效、诚信、廉洁政府，认真落实全面从严治党各项要求，扎实推进“两学一做”学习教育常态化制度化，为民务实清廉的从政理念在政府系统进一步强化。不断增强“四个意识”，提高政治站位，确保上级党委政府的指示要求和市委的决策部署落到实处。坚持向人大报告、政协通报工作制度，提案议案办结率达到100%。注重运用法治思维和法治手段化解矛盾，市县乡三级政府法律顾问制度全面建立，82%的乡镇(街道)建成公共法律服务站。深化放管服改革，市县行政权力事项分别压减34%和27%，涉企营业执照实现“三十一证合一”和全程电子化登记，市场主体活力进一步增强。积极办好政务服务热线，群众诉求事项办结率达90%以上。强化行政监察和审计监督，扩大政务公开，不敢腐不能腐不想腐的机制正在形成。妇女儿童、人民防空、民族宗教、统计、物价、史志、档案、外事、侨务、贸促会、红十字会、防震减灾等工作受到省级以上表彰，国防动员、民兵预备役、双拥共建、对台、气象、保密、慈善、应急管理和对口支援等工作都取得新的成绩。

各位代表，困难中的转型来之不易，压力下的收获更为可贵。一年来，面对结构调整深化期，我们主动适应新常态，以奋发进取的“上行动力”应对宏观经济的“下行压力”，增创了经济发展新优势；面对矛盾风险积聚期，我们坚守红线底线，以壮士断腕的气魄，打出了环境保护、安全生产、节能减排“组合拳”，闯出了规范发展、绿色发展的新路子；面对改革创新攻坚期，我们以勇于

担当的精神克难前行、团结奋进，凝聚了“走在前列”的强大合力。

各位代表，去年的发展成绩实属不易、令人鼓舞。这是省委、省政府和市委正确领导的结果，是市人大、市政协监督支持的结果，是全市人民拼搏奋斗和各方面关心帮助的结果。在此，我代表市人民政府，向全市广大干部群众，向人大代表、政协委员、各民主党派、工商联、人民团体和离退休干部，向驻临人民解放军、武警官兵和中央、省属部门单位，向所有支持临沂发展的各界朋友，表示衷心的感谢和崇高的敬意！

在肯定成绩的同时，我们也清醒地认识到，当前我市发展不平衡不充分的问题还比较突出：人均占有偏低，经济大而不强、大而不优、大而不均的状况没有根本改变；传统产业层次不高，新兴产业发展缓慢，企业创新能力薄弱，新动能尚未成为主导，转型升级还在路上；县域经济整体实力不强，基础设施、公共服务体系还不完善，城乡一体化发展任重道远；环境、资源等要素制约持续趋紧，各类风险隐患不断显现，守住底线需要付出艰辛努力；政府工作人员作风不够严实，放管服改革没有完全到位，基层群众和企业不满意、不高兴的事还有很多，等等。对此，我们一定高度重视，采取更加有力、更为精准的措施，尽快加以解决。

二、2018年工作安排

今年是全面贯彻党的十九大和省第十一次党代会精神的开局

之年，是改革开放40周年，也是决胜全面建成小康社会、实施“十三五”规划承上启下的关键一年。进入新时代，推进新发展，我们信心满怀。党的十九大科学描绘了未来发展的宏伟蓝图，为我们向新的奋斗目标前进指明了方向。当前，全球经济持续复苏，我国经济已由高速增长转向高质量发展，宏观经济形势对我市转型发展十分有利。经过长期不懈努力，全市经济社会发展综合实力不断增强，城市形象大幅提升，干部群众自信心自豪感进一步提振。只要我们用好机遇、发挥优势，紧盯目标不懈怠、坚定不移抓落实，就一定能够夺取“大美新”临沂建设的新成就！

今年政府工作的总体要求是：深入贯彻党的十九大、中央经济工作会议精神和上级决策部署，按照“走在前列”的目标定位，坚持以稳中求进为工作总基调、以高质量发展为根本要求、以供给侧结构性改革为主线，突出抓好新旧动能转换、产业转型升级、双向开放拓展、城乡协调发展、风险防范化解、民生福祉提升和政府自身建设，加快解决发展不平衡不充分的问题，奋力开创新时代临沂加快发展、创新发展、高效发展、绿色发展的新局面。

今年工作的主要目标是：地区生产总值增长8%左右，一般公共预算收入增长7%左右，固定资产投资增长10%左右，社会消费品零售总额增长10%左右，进出口总额保持稳定增长，城乡居民人均可支配收入分别增长8%和8.5%左右，城镇登记失业率控制在3.5%以内，全面完成省政府下达的年度节能减排降碳约束性指标。

做好今年的工作，需要牢牢把握好以下四条原则：

一是坚持把习近平新时代中国特色社会主义思想贯穿政府工作始终。自觉用习近平新时代中国特色社会主义思想武装头脑、指导实践、推动工作，牢固树立战略思维、系统思维、创新思维和底线思维，正确处理强自信与戒自满、抓发展与保稳定、借外力与活内力、想干事与会干事、守纪律与敢担当的关系，主动把本地区本部门工作置于全国全省大局下摆布，以世界眼光、国际标准、沂蒙特色为标杆，进一步解放思想、创新举措，推动十九大精神在沂蒙大地开花结果。二是坚持把发展作为解决一切问题的基础和关键。立足发展质量不高、综合实力不强的最大市情，坚持“一切围绕发展、一切促进发展、一切保障发展”，加快补齐短板，着力推动经济发展更高质量、更有效率、更加公平、更可持续。三是坚持把守住底线作为发展的根本保障。高度重视和化解各类风险隐患，以最高标准、最严要求、最硬措施，坚决打好安全生产、生态环保、社会稳定、金融风险防控和精准脱贫五场攻坚战，进一步巩固和谐稳定的社会局面。四是坚持把为人民谋福祉作为最大追求。牢固树立以人民为中心的发展思想，注重从群众最关注的事情做起、从群众最不满意的事情改起，带着感情和责任办好民生实事，努力让老区人民享有更多的获得感。

今年的政府工作，突出抓好以下六个方面：

(一)加快转换新旧发展动能。以制定规划、建立项目库、设立引导基金、完善推进机制为重点，培育新动能，推动新发展。抓好项目带动。重点实施100个“四新四化”项目、培育100家高成长性企业、搭建100个产业创新平台，打造一批优势产业和骨干企业。全面落实“一个项目、一个专班”的工作方式，推动政策向项目集成、资源向项目集聚、人才向项目集中。强化创新驱动。充分发挥企业主体、园区科技平台孵化、创新人才带动和对外科技合作促进四个方面的作用，提高区域科技创新实力。制定科技创新及人才引进的优惠政策，鼓励企业与高等院校、科研院所共建实验室、研究中心、技术中心和海外研发中心。建立创新成果要素交易市场，建设科技服务中心，健全科技创新成果转化机制。加强知识产权创造、运用和保护，依法甄别社会反映强烈的产权纠纷案件，培育一批特色鲜明、协同发展的创新型企业。深化改革推动。开展“全面深化企业改革突破年”活动，完善以“管资本”为主的国资监管模式，全面推行现代企业制度，积极发展混合所有制经济。加大中小企业服务体系和信用担保体系建设力度，全面实施并不断完善市场准入负面清单制度，充分发挥各类商会、协会在联系政府、企业和市场中的桥梁纽带作用，促进非公有制经济发展壮大。支持高新区、经开区创新机制，尽快成立集中统一的行政审批机构，实现“一个主体对外、一枚印章审批、一个平台服务”。抓好“证照分离”改革试点，以机制创新、效能提升激发园区活力，打造新旧动能转换示范区。深化放管服改革，再削减一批行政权力事项、取消一批证明和盖章类材料、推出一批“整链条”办理事项。加快政务信息系统整合共享，扩大“互联网+政务服务”，促进实体和网上“两个政务大厅”深度融合。科学配

置市场要素，放活土地二级市场，推进公共资源公开公平交易，运作好新旧动能转换基金，助推经济发展质量变革、效率变革和动力变革。

(二)加快建立现代产业体系。以高质量发展为目标，实施“8+8”现代产业培育计划，做优食品、木业、机械、冶金、化工、建材、医药、纺织服装八个传统产业，培育电子商务、智慧物流、农业新六产、文化创意、全域旅游、医养健康、智能制造、数字经济八个新兴产业。实施工业转型攻坚行动。把处置“僵尸企业”作为重要抓手，运用法治思维、市场办法和行政手段，淘汰一批小钢铁、小化工、小建陶等落后产能。改造提升传统产业，争取木业、建陶、五金、化工等产业年内有过半企业完成升级改造。认真落实税费减免政策，从严整治乱收费现象，推动企业降本增效。开展中小企业“四个一百”示范工程，鼓励企业对标赶超。积极支持企业直接融资，力争年内新增上市和挂牌企业10家以上。加强企业家队伍建设，抓好企业家培训，弘扬企业家精神，构建亲清新型政商关系。实施服务业创新发展行动。瞄准消费升级方向，大力发展信息科技、金融服务、法律服务等生产性服务业。依托红色资源、地域特色，积极发展艺术品交易、影视创作等文化产业。办好2018年中俄红色旅游合作交流系列活动，进一步打响“亲情沂蒙·红色临沂”品牌。实施农业提质增效行动。以优化农业产能和增加农民收入为目标，以品牌农业、生态农业、设施农业、休闲农业、智慧农业为发展方向，依托临沂国家农业科技园

区、兰陵国家农业公园等平台，抓好一批农业“新六产”项目，示范引领全市农业生产集约、经营专业、三产融合。全面落实农村土地所有权、承包权、经营权“三权”分置办法，深化农村集体产权制度改革，积极培育新型农业经营主体，建设一批田园综合体，促进小农户与现代农业发展有机衔接。推进“大数据+农业”，支持发展农村电商，建设农博馆和农产品展销平台，办好永不落幕的农博会。实行粮食安全和“菜篮子”工程行政首长负责制，确保主要农产品供给。加强农村饮水安全、水库堤坝除险加固和农田水利设施建设，抓好农药、化肥减量增效，增强农业可持续发展能力。实施“品牌临沂”建设行动。倡树工匠精神，扶持骨干企业、优势产业和县域提高品牌质量，打造新品牌、保护老品牌、打击假冒品牌，提升临沂品牌整体形象。

(三)加快形成开放型经济格局。以商城国际化为引领，深度融入“一带一路”建设，用好国际国内两种资源、两个市场，全方位、多层次、宽领域扩大对内对外开放。全力打造国际商贸城核心区，发展金融供应链、服务外包、云计算等新型业态，建好现代物流、智慧产业、跨境贸易等特色项目。大力推进老商贸城改造，重点抓好林丰国际物流城、华强建材城等老市场的提质增效。规范发展网上商城，推行跨境电商、垂直电商、电商服务商、网商一体化发展模式，建成运营市级电子商务公共服务中心，争创跨境电商综合试验区和电子商务示范基地。科学布局建设“海外临沂商城”，提高中欧商贸物流园等海外商城招商运营水平，

沿“一带一路”节点城市再布局一批海外商城。加强综合保税区和临沂商城的业务衔接，推广国际贸易“单一窗口”，放大“境内关外”政策优势，招引一批高端高质产业项目，建立商城国际化新窗口。用好“市场采购”贸易方式试点政策，吸引更多企业和境外采购商到临沂采购物资，建设具有国际影响力的工程物资集采集供基地。继续办好商博会、木博会、食博会和五金商品交易会等特色展会，鼓励企业走出去参加境外展会。以现代物流园区建设为抓手，打造综合公路交通枢纽，规范提升公路物流。推进鲁南铁路物流园区建设，按照“五定”标准开行中欧货运班列。加密国内货运航线，开通国际货运航班。完善临沂港功能，建设国检试验区。发挥综合保税区海关特殊监管区域的作用，发展保税仓储物流。鼓励商贸企业向产销一体、内外并举转化，实现由集散型向产地型、内贸型向外向型转变。在各类开发区和经济园区内，建立面向临沂商城的地产品加工专业区，发展外贸优势产业集群。支持商城外贸企业采取“代加工”方式，与上游企业建立稳定的合作关系，全面提高地产品加工业规模和水平。坚持“走出去”与“引进来”结合，瞄准重点区域、优势产业、龙头企业，开展产业招商、专业招商、企业招商，大力招引“四新四化”项目。继续深化与港澳台等地经贸合作，组织骨干企业参加香港山东周、鲁台经贸洽谈会等活动，积极开拓国际市场，促进全市进出口贸易和实际利用外资稳定增长。

(四)加快推动城乡一体化发展。以中心城区为龙头，促进

城乡联动、优势互补，全面提升城镇化发展质量。年内，完成18万农业转移人口市民化，户籍和常住人口城镇化率分别达到48.6%和59%。开展新一轮城市总体规划修编，抓好北城新区二期开发，启动高铁片区建设，推进西部新城、东部生态城、南部循环经济区开发和莒南撤县设区，拓展中心城区“北上、东进、南优、西强”发展空间。发展综合立体交通，加快鲁南高铁、京沪高速改扩建和新台、岚临高速及机场新航站楼建设，做好京沪高铁二通道、济莱临高铁和张临、董梁高速等前期工作，创建全国综合运输服务示范城市。发挥滨水优势，做活水文章，贯通水系、净化水体、提升水景，彰显“九水绕城、城水相依”的城市特色。完善城市功能，抓好新党校、体育中心等一批重点工程，启动中心城区第二水源地建设，新建海绵城市25平方公里、综合管廊12公里、装配式建筑80万平方米，新增供热面积300万平方米、燃气管网80公里。实施新一轮县域经济振兴计划，按照主体功能区定位，放眼全国全省对标赶超，在全市域布局县区主导产业，落实“飞地”政策，支持县区共建合作园区，打造特色鲜明、竞争力强的产业集群。创新财税激励机制，稳步推进市以下收入划分、财政事权和支出责任划分等配套改革，调动县域加快发展、扩大财源的积极性。用好新型城镇化、中小城市试点政策，加快沂水、平邑、郯城三个市域副中心建设，支持沂南、临沭、费县、兰陵打造中心城区卫星城，扶持蒙阴建设生态功能区。鼓励乡镇完善道路、供水、供电、通信、物流等基础设施，特色化发展农产品精深加工、民

俗旅游、体育休闲、健康养老等产业，建设一批产业集聚型、创新创业型、文化传承型特色小镇。科学制定乡村振兴战略规划，统筹布局现代农业产业体系、生产体系、经营体系，引领农业成为有奔头的产业、农民成为有吸引力的职业、农村成为安居乐业的家园。健全城乡融合发展体制机制，积极吸引工商资本下乡、农村人才留乡、成功人士回乡创业，培育一批懂农业、爱农村、爱农民的“三农”工作队伍。完善农村基础设施，提升“四好农村路”水平，实施“厕所革命”新三年计划，加强特色村落保护，建设一批生态观光型、民俗民宿型、农事体验型“美丽乡村”，推动农村产业兴旺、生态宜居、乡风文明、治理有效、生活富裕。

(五)加快防范化解各类风险隐患。以打赢五场攻坚战为抓手，着力推动经济社会健康协调可持续发展。打好精准脱贫攻坚战。注重扶贫同扶志结合，增强产业扶贫、金融扶贫、搬迁扶贫实效，年内再减贫1.8万人，基本完成脱贫任务。完善脱贫长效机制，推进“四联八建”贫困村提升，设立动态性返贫人口扶贫基金，加强扶贫资金资产和产业项目管理，确保扶贫项目有效运营、集体资产长期收益。扎实推进雨露计划、“三无”失能贫困人口救助、扶贫特惠保险等项目，保证现行标准下的脱贫质量。打好生态环保攻坚战。切实抓好中央环保督察反馈问题整改，加强生态文明建设目标评价考核，深入实施“气十条”“水十条”和“土十条”，建立市场化、多元化生态补偿机制，大力发展绿色低碳经济、生态环保产业，严格节能减排考核奖惩，坚决守住生态功能保障基

线、环境质量安全底线和自然资源利用上线。调整工业产业布局，优化交通、用能结构，狠抓工业、散煤、畜禽养殖和扬尘等污染治理，将大气污染防治进行到底，坚决打赢蓝天保卫战，还群众一片蓝天。严格落实河长制，抓好河流断面水质改善、黑臭水体治理，实施“放鱼养水”工程，还群众一河清水。强化土壤污染治理，建设一批危废处置项目，提高全过程处置能力，还群众一方净土。积极响应“国土绿化”行动，持续实施“绿满沂蒙”计划，建立矿山环境治理恢复基金制度，抓好环城生态防护林带、荒山绿化和绿色通道提升工程，创建沂蒙山世界地质公园，还群众一山绿色。打好安全生产攻坚战。深刻汲取金誉石化“6·5”爆炸着火事故的沉痛教训，全面落实安全生产责任制，进一步完善风险分级管控和隐患排查治理双重预防体系，加强安全生产管理和全员培训，抓好危化品、非煤矿山、道路交通、建筑施工、消防等重点行业和领域综合整治，确保安全生产形势持续稳定好转。打好防范化解金融风险攻坚战。聚焦担保圈、债券违约、互联网金融等风险问题，健全“政府牵头、企业自救、银行配合、政监银企密切协作”的风险防控机制，严厉打击非法吸收公众存款、发放高利贷、集资诈骗、恶意逃废债务等违法犯罪活动，持续优化金融生态。深化金融改革，发展普惠金融，促进金融与实体经济、金融与房地产、金融体系内部的良性互动、共赢发展。规范政府举债融资行为，妥善化解存量债务，严控新增债务。打好维稳攻坚战。深化基层平安创建活动，及时解决影响群众安全和社会稳定的突

出环境问题。坚持领导干部公开接访、包保重点疑难信访案件，健全人民调解工作体系，完善社会矛盾排查预警和多元化解机制，实现基层基础网格实体化、管理员专职化，促进信访问题就地化解。继续实施公安基层基础建设三年计划，抓好“雪亮工程”延伸应用，筑牢社会治安立体化防控体系。织牢公共法律服务网络，全面实行“一村一法律顾问”，努力让群众获得及时有效的法律服务。

(六)加快提高群众幸福感和满意度。以办好十大工程百项惠民实事为切入点，全面落实“七有”要求，努力让改革发展成果更多更公平地惠及千万群众。解决结构性就业矛盾，构建城乡统一的就业援助制度，大力开展职业技能培训，突出抓好高校毕业生、失业人员、城镇困难人员、退役军人等重点群体就业创业。进一步完善社会保障制度，实施全民参保计划，抓好职工长期护理保险制度试点，低保补助标准再提高13.5%。全面推进惠民殡葬改革，降低丧事成本，减轻群众负担。推动教育扩容提质，实施校舍建设“双百工程”，新建改扩建中小学、幼儿园各100所，统筹抓好化解“大班额”、全面改薄、中小学生暖冬、智慧教育普及等工作。积极构建配置合理、平价优质的早教托育服务体系，加快解决婴幼儿照护问题。支持高等院校、职业院校和特殊教育发展，建立终身教育体系。提升医疗服务水平，启动市人民医院东城医院和北城医院医养中心、市中医院北城医院等重点医疗项目建设，实施中医药振兴、“百镇千村”医疗卫生服务提升计划，全

面推进分级诊疗和“两医两化”建设。发展慈善事业，健全农村留守儿童和妇女、老年人关爱服务体系，抓好残疾康复服务。加强商品房市场调控，搞好棚户区、老旧小区改造，开展物业行业文明创建，完善多主体供给、多渠道保障、租购并举的住房制度，支持合理住房消费，抑制投资投机行为。优化城乡路网和公交线网布局，注重停车设施规划建设和运营管理，缓解交通拥堵压力。启动“国家历史文化名城”创建工作，推进文体惠民工程，办好书圣文化节、惠民文化消费季、全国马拉松等节会活动，更好地满足群众文化和健身需求。依托各类红色文化资源，建设爱国主义、党性党史和廉政教育基地。实行食品药品安全源头严防、过程严管，把好群众饮食用药安全关口，实现学校食堂、旅游景区餐饮单位监管和食品药品安全审稽全覆盖，创建省级食品安全先进市。加强对人口生育的政策引导和技术服务，提高出生人口素质。抓好气象、地震、人防、消防、应急管理等工作，提升防灾减灾救灾能力。深化政务、村务、厂务公开，扩大基层民主。深入践行社会主义核心价值观，扎实开展文明创建、双拥共建、道德模范评选、乡村移风易俗等活动，树立新时代沂蒙新风尚。

三、关于政府自身建设

各位代表，新时代孕育新机遇，新征程呼唤新作为。全面完成今年的政府工作任务，要求各级政府工作人员必须大力弘扬沂

蒙精神，凝心聚力、团结实干，忠实履行引领发展、服务群众、共建和谐的神圣职责。

要不断提高政治站位。扎实开展“不忘初心、牢记使命”主题教育，切实增强政治素养和党性觉悟。进一步树牢“四个意识”，坚决在思想上政治上行动上同以习近平同志为核心的党中央保持高度一致，做到思想上充分信赖、政治上坚决维护、组织上自觉服从、感情上深刻认同、行动上始终跟随。坚定“四个自信”，准确把握习近平新时代中国特色社会主义思想内涵，紧密结合实际创造性开展工作，时刻忠诚于党和人民的事业。坚决落实“四个服从”，不折不扣地贯彻执行党中央国务院、省委省政府和市委的各项决策部署，确保令行禁止、政令畅通。

要全面加强能力建设。提高科学发展的能力，大兴调查研究之风，深入基层一线，在学思践悟笃行中提高专业素养。坚持功成不必在我、一张蓝图绘到底，面对前进中的矛盾和问题不推诿、不避让、不退缩，敢挑最重的担子，敢啃最硬的骨头，努力创造经得起群众和时代检验的业绩。提高群众工作的能力，自觉践行群众路线，深化“结亲连心”活动，推动机关干部深入基层、服务群众。提高处理各种复杂矛盾的能力，保持攻坚克难的作风韧劲，勇于改革创新，敢于打破路径依赖，牢牢把握工作主动权。提高抓落实的能力，不断完善正向激励、考核评价、容错纠错和澄清保护机制，旗帜鲜明地为勇于担当、踏实做事、不谋私利的干部撑腰鼓劲。对基层干部既要从严要求、又要松绑减压，做到政治上关心、工作上支持、生活上帮助、成长上爱护，激励广大基层干部敢于负责、大胆开拓、干事创业。

要大力促进公平正义。牢固树立依法行政理念，坚持宪法至上，信仰法律、尊崇法律、践行法律，把政府一切活动纳入法治化轨道。严格规范政府重大决策行为，认真落实公众参与、专家论证、合法性审查和集体讨论决定等制度，邀请相关利益人参加市政府有关会议，提高依法科学民主决策水平。强化政府层级监督，依法查处违法或不当行政执法行为，促进公正文明执法。发挥政府诚信的导向作用，搭建信用评估体系和公示平台，加快涉企信息统一归集公示，实施信用协同监管和联合惩戒，真正使守信者得益、失信者受罚。深入推进政务公开，自觉接受人大法律和工作监督、政协民主监督，加强政府内部监督，主动接受社会和舆论监督，确保政府权力在阳光下运行。

要从严从实正风肃纪。认真履行政府系统全面加强党的建设、全面从严治党主体责任，严格执行中央八项规定实施细则和省、市配套办法。全面落实办公用房、公务用车等新要求，自觉抵制公款吃喝、公款送礼、公款旅游等行为，着力在改进调查研究、精文简会、规范公务接待、厉行勤俭节约等方面见到新成效，持之以恒地反对和纠正形式主义、官僚主义、享乐主义和奢靡之风，始终保持艰苦奋斗的优良传统。强化纪律执行，让各级干部知敬畏、存戒惧、守底线，自觉在严监督、紧约束条件下创造性地开展工作。加强审计监督，完善统计体制，制止一切形象工程、政

绩工程，反对任何数字造假。始终保持反腐高压态势，自觉接受监察委员会的监督，坚决纠正损害群众利益的行为，巩固不敢腐的氛围、完善不能腐的制度、构筑不想腐的堤坝，打造一支绝对忠诚、干事担当、干净自律、充满活力的人民公仆队伍。

各位代表，进入新时代，站在新起点，我们肩负着光荣的历史使命，前景光明，任务艰巨，责任重大。让我们紧密团结在以习近平同志为核心的党中央周围，在省委、省政府和市委的坚强领导下，不忘初心、牢记使命，团结拼搏、开拓奋进，为建设“大美新”临沂、谱写新时代中国特色社会主义沂蒙篇章而努力奋斗！
